# Tunable Electronic, Optical, and Thermal Properties of two- dimensional Germanene via an external electric field

**DOI:** 10.1038/s41598-020-57558-x

**Published:** 2020-01-20

**Authors:** Raad Chegel, Somayeh Behzad

**Affiliations:** 1 Physics Department, Faculty of Science, Malayer University, Malayer, Iran; 2 Department of Engineering Physics, Kermanshah University of Technology, Kermanshah, Iran

**Keywords:** Nanoscale materials, Electronic properties and materials, Optoelectronic devices and components, Optical properties and devices

## Abstract

In this paper, we present a tight-binding model based on DFT calculations for investigation the electronic and optical properties of monolayer Germanene. The thermal properties are investigated using Green function method. The required tight binding parameters including the onsite energies and third nearest neighbors hopping and overlap integrals are obtained based on our DFT calculations. Germanene is a semiconductor with zero band gap and linear band dispersion around the K point. The band gap opening occurs in the presence of bias voltage. The band gap is increased linearly with increase of the bias voltage strength. The tight binding results for position of the two first peaks in the optical Infrared region is same with the DFT results. By applying and increasing bias voltage, the dielectric function shows the blue shift by reduction the peak intensity in the energy range E < 1 eV. The thermal conductivity and heat capacity increase with increasing the temperature due to the increasing of thermal energy of charge carriers and excitation them to the conduction bands. The thermal properties of Germanene in the absence of bias U = 0 is larger than that U ≠ 0 and they decrease by further bias strength increasing, due to the increasing band gap with bias.

## Introduction

Two-dimensional (2D) monolayer structures have been an active field of research due to their electronic, optical, and thermal properties. Graphene is a planar atomic layer of carbon atoms which they are arranged in a honeycomb lattice with 1.42 A° interatomic distance between two adjacent C atoms. Graphene has been an active field of research due to its important physical properties such as electronic structure with gapless properties, room temperature quantum Hall effects^1^ and high intrinsic mobility^2^.

In the graphene, the existence of a zero gap is due to the crossing between the valence and conduction bands at the K point (Dirac point). Near the K point, graphene exhibits linear energy dispersion in terms of momentum and its charge carriers behave as relativistic massless Dirac fermions described by a Dirac-like equation^3^. Note that, Materials with a zero band gap energy exhibit some fascinating and superior electronic properties compared to materials with a non-zero energy gap and they have intriguing physical properties and numerous potential practical applications in spintronics, electronics, optics and sensors^4^. The Dirac Spin-gapless semiconductors, have high carrier mobility and due to their real massless fermions and dissipation-less transport properties, they are regarded as promising candidates for applications in ultra-fast and ultra-low-power spintronic devices^5,6^.

Several studies on electronic and optical properties of monolayer graphene have been reported via Density functional Theory (DFT) and Tight binding Theory (TB)^7–11^. Due to the zero band gap in graphene, the light absorption occurs in a wide range of spectra from infrared (IR) to ultraviolet which lead to the use of graphene in electro-optical devices. Using the first nearest neighbor tight binding model, the electronic structure of graphene can be found analytically but this model does not accurately reproduce the π and π* graphene bands over a sufficiently large range of the Brillouin zone^12^. This model can be completed in agreement with DFT results by adding more parameters such as third and fifth next-neighbors hopping and overlap integrals^12,13^. The successful results in the study of graphene have expanded the scientific interest on studying the theoretical and experimental of other elements such as Si and Ge atoms with similar two-dimensional stable honeycomb structures which referred to as Silicene (Si) and Germanene (Ge)^14^. In contrast to planar graphene, the planar Germanene is unstable and becomes stable with small buckling (∆0 = 0.64 Å), due to the mixing of sp2 and sp3 hybridization^15,16^ which ∆0 is defined as the vertical distance between the two planes of atoms. Due to the existence of buckling distance, the band gap of Germanene can be controlled by applying an external electric-field along the vertical axis^16^. Due to large buckled distance, the intrinsic carrier mobility in Germanene is higher than graphene^17^. The electronic properties of graphene and Germanene are similar because of their same hexagonal symmetry. Similar to graphene, the Germanene shows linear dispersion relation with valence band (VB) and the conduction band (CB) which crossing at the K and K’ points of the Brillouin zone^18^. It was predicted by first-principles calculations that the spin–orbit interaction in Germanene leads to small band gap opening at the Dirac point and unlike to massless fermions in graphene, the Germanene has massive Dirac fermion^19,20^. The band gap opening due to the spin orbit coupling for Germanene is higher than graphene^21^. The Fermi velocity decreases along the row C, Si and Ge, independent of the functional^17^. It is found that the Fermi velocities in the vicinity of the Dirac point is about 6.3, 5.1 and 3.8 (×10^5^ m/s) for graphene, Silicene, and Germanene, respectively^22^. The intrinsic carrier mobility of Germanene is higher than graphene and Silicene^17^.

Using ab initio calculations, it was found that an external electric field is able to produce a tunable band gap in single-layer buckled Germanene and the band gap increases linearly with the electric field strength^23^.

Furthermore, the existence of different dopants within the monolayer Germanene causes the sizable band gap opening at the Dirac point^24,25^ and affects the electro-optical properties of this material. Theoretical calculations have shown that Germanene can act as good gas sensors for N2, NH3, NO2, NO and O2 gases^26,27^. Also, Germanene is expected to show a higher surface reactivity compared to graphene due to its buckled structure.

Many theoretical studies have been done to understand the mechanical and thermal properties of Germanene^16,21,28–30^. Unlike to biaxial strain, the small uniaxial strain or vertical electric field creates a direct gap at K point in Germanene and the value of band gap increases with the increase of uniaxial strain or vertical electric field^30^. By investigation the phonon spectrum, it was found that the Germanene lattice is stable by applying and increasing the strain up to 16% and in this range the Dirac cone shifts towards higher energy^31^.

Similar to Graphene, the optical properties of Germanene have been also interested in the recent years^10,32^. By studying their optical properties, it has been found that the optical response is shifted from ultraviolet to infrared from graphene to Ge^29^. The optical properties calculations revealed that the Germanene sheet has significant light absorption of solar spectrum and shows optical anisotropy^30^.

The aim of the present article is to provide a tight binding model and Green function approach based on the DFT results, for investigation the electrical, optical and thermal properties of monolayer Germanene. Finding a suitable hopping integrals and onsite parameters is of crucial importance for comparison of tight binding results with results from DFT studies. In this work, based on first-principles calculations, we obtained the required tight binding parameters, by considering the third nearest neighbor overlap and hopping integrals. Using our obtained parameters, the results for electronic structure such as linear bands around the K-point are in good agreement with the DFT calculations. The tight binding calculation for optical spectrum of Germanene has not been performed until now, so investigation the tight binding optical spectrum is one of the interesting steps in this paper. We calculated the optical dielectric function of Germanene by calculating the dipole matrix elements and compare the results with DFT calculations. Finally, using the suitable hopping integrals and onsite energies, the thermal properties of monolayer Germanene investigated using the tight binding model and Green function approach based on Kubo-Greenwood formula.

The paper is organized as follows. The tight binding formalism and DFT computational details are presented in sections 2 and 3. The results and discussions including the electronic, dipole matrix elements, optical and thermal properties, are discussed in Sec. 4.

## Tight Binding Formalism

### Electronic structure

An analytical formula using first nearest neighbor tight binding (1NN-TB) model, has been found for calculation of π-band structure of graphene layer^12^. By adding the next-neighbor hopping integrals and overlap, the tight binding model will be accordant with the DFT results. In this section the 3NN-Tight binding model has been used to obtain the Hamiltonian (H) and overlap (S) matrix elements. The Bravious lattice site of all systems included two atoms the wave function ${\Psi }_{l}({\boldsymbol{k}},{\boldsymbol{r}})$ can be written in terms of Bloch functions ${\Phi }_{\alpha }({\boldsymbol{k}},{\boldsymbol{r}})$ for the two nonequivalent atoms (α = A, B) in the unit cell as:

$${\Psi }_{l}(k,r)={C}_{A}^{l}({\boldsymbol{k}}){\Phi }_{A}({\boldsymbol{k}},{\boldsymbol{r}})+{C}_{B}^{l}({\boldsymbol{k}}){\Phi }_{B}({\boldsymbol{k}},{\boldsymbol{r}})$$

where the ***k*** is the wave vector, ${C}_{s}^{l}(k)$ is the expansion coefficients and $l=c,v$ shows conduction/valance band. The Bloch function in terms of the orbital wave function is written as:

$${\Phi }_{\alpha }({\boldsymbol{k}},{\boldsymbol{r}})=\frac{1}{\sqrt{N}}\,\sum _{{{\boldsymbol{R}}}_{\alpha }}\,{e}^{i{\boldsymbol{k}}.{{\boldsymbol{R}}}_{s}}\,{\phi }^{(\alpha )}({\boldsymbol{r}}-{{\boldsymbol{R}}}_{\alpha })$$

where ${{\boldsymbol{R}}}_{\alpha }$ is the position of the α atom in the unit cell, ${\phi }^{(\alpha )}$ is the atomic orbital in the unit cell and N is the number of the unit cell. By solving the Schrodinger equation, the band structure E(k) and ${C}_{\alpha }^{l}({\boldsymbol{k}})$ coefficients are obtained as,

$$(\begin{array}{cc}{H}_{AA}({\boldsymbol{k}}) & {H}_{AB}({\boldsymbol{k}})\\ {H}_{BA}({\boldsymbol{k}}) & {H}_{BB}({\boldsymbol{k}})\end{array})(\begin{array}{c}{C}_{A}^{l}({\boldsymbol{k}})\\ {C}_{B}^{l}({\boldsymbol{k}})\end{array})={E}^{l}({\boldsymbol{k}})(\begin{array}{cc}{S}_{AA}({\boldsymbol{k}}) & {S}_{AB}({\boldsymbol{k}})\\ {S}_{BA}({\boldsymbol{k}}) & {S}_{BB}({\boldsymbol{k}})\end{array})(\begin{array}{c}{C}_{A}^{l}({\boldsymbol{k}})\\ {C}_{B}^{l}({\boldsymbol{k}})\end{array})$$

The Hamiltonian and overlap matrix elements [H_αβ_ and S_αβ_] describe the coupling between two neighbor atoms on α and β sites and can be expanded in term of the transfer ${\gamma }_{i}$ and overlap ${s}_{i}$ parameters. The diagonal elements ${H}_{\alpha \alpha }$ and ${S}_{\alpha \alpha }$ describe the coupling between two atoms both on α site and have the coupling to it self and its 2-st nearest neighbors, while the off-diagonal ones represent the coupling between α and β sites and have the coupling to 1-st and the 3-st nearest neighbors. The matrix elements are given by

$$\begin{array}{rcl}{H}_{\alpha \alpha }({\boldsymbol{k}}) & = & {\varepsilon }_{\alpha }+{\gamma }_{2}{F}_{2}({\boldsymbol{k}})\\ {H}_{\alpha \beta }({\boldsymbol{k}}) & = & {\gamma }_{1}{F}_{1}({\boldsymbol{k}})+{\gamma }_{3}{F}_{3}({\boldsymbol{k}})\\ {S}_{\alpha \alpha }({\boldsymbol{k}}) & = & 1+{s}_{2}{F}_{2}({\boldsymbol{k}})\\ {S}_{\alpha \beta }({\boldsymbol{k}}) & = & {s}_{1}{F}_{1}({\boldsymbol{k}})+{s}_{3}{F}_{3}({\boldsymbol{k}})\end{array}$$

The hopping integrals and overlap parameters are

$$\begin{array}{rcl}{\varepsilon }_{\alpha } & = & \langle {\varphi }_{\alpha }({\boldsymbol{r}}-{R}_{\alpha })|H|{\varphi }_{\alpha }(r-{R}_{\alpha })\rangle \\ {o}_{2} & = & \langle {\varphi }_{\alpha }({\boldsymbol{r}}-{{\boldsymbol{R}}}_{\alpha })|H|{\varphi }_{\alpha }({\boldsymbol{r}}-{{\boldsymbol{R}}}_{\alpha }-{{\boldsymbol{d}}}_{2})\rangle \\ {o}_{1,3} & = & \langle {\varphi }_{\alpha }({\boldsymbol{r}}-{{\boldsymbol{R}}}_{\alpha })|H|{\varphi }_{\alpha }({\boldsymbol{r}}-{{\boldsymbol{R}}}_{\alpha }-{{\boldsymbol{d}}}_{1,3})\rangle \end{array}$$

where *o* ≡ *γ*, *s*, ${F}_{m}({\boldsymbol{k}})={\sum }_{{{\boldsymbol{d}}}_{m}}\,{e}^{-i{\boldsymbol{k}}.{{\boldsymbol{d}}}_{m}}$ and **d**_1_, **d**_2_ and **d**_3_ correspond to the distances to first, second and third neighbors, respectively. The hopping integral ${\gamma }_{i}$ and overlap ${s}_{i}$ parameters are btained by fitting the tight binding results to the density functional theory. Each $\alpha$ atom has three first (${{\bf{d}}}_{1}$) and third (${{\bf{d}}}_{3}$) neighbor atoms with different sublattices $\beta$ and distances ${b}_{0}$ and $2{b}_{0}$, respectively. The second neighbors (${{\bf{d}}}_{2}$) are in the same sublattice $\alpha$ and distance $\sqrt{3}{b}_{0}$. The relative vector for ${{\bf{d}}}_{m}$ for first to third nearest neighbor atoms are:

$$\begin{array}{rcl}{{\bf{d}}}_{1} & \equiv  & {b}_{0}(1,0),{b}_{0}(\,-\,1,\pm \,\sqrt{3})/2\\ {{\bf{d}}}_{2} & \equiv  & \pm {b}_{0}(0,\sqrt{3}),\pm \,{b}_{0}(\,-\,3,\sqrt{3})/2,\mp \,{b}_{0}(3,\sqrt{3})/2\\ {{\bf{d}}}_{3} & \equiv  & {b}_{0}(2,0),{b}_{0}(1,\mp \,\sqrt{3})/2\end{array}$$

We give our determined tight binding parameters ${\gamma }_{i}$ and ${s}_{i}$ for Germanene in Table 1. Our parameters give the correct band gap in comparison with our DFT calculation and other studies. By using the electronic band structure ${E}^{c,v}({\boldsymbol{k}})$, the density of sate (DOS) can be calculated as^33^:

$$D(\omega )=\frac{{N}_{f}}{{(2\pi )}^{2}}{\int }^{}\,\sum _{m}\,\delta (\omega -{E}^{m}({\boldsymbol{k}}))d{\boldsymbol{k}}$$

where ${N}_{f}$ is a degeneracy factor and the **k** integral is over a region surrounding the first Brilouan zone. The DOS exhibits prominent peaks due to the band edge of sub-bands where the peak positions correspond to the band edge state energies. The DOS exhibits prominent asymmetric peaks due to the band edge of parabolic subbands. The DOS peak positions are related to the band edge state energies and the DOS heights are proportional to inverse square root of the subband curvature and band degeneracy.

**Table 1:** The 3NN- hopping and overlap Tight binding parameters for Germanene.

Structure	*γ*_1_	*γ*_2_	*γ*_3_	*s*_1_	*s*_2_	*s*_3_
Ge	−1.163	−0.055	−0.0836	0.01207	0.0128	0.048

The optical matrix element of possible transitions between the initial [${\Psi }^{i}$] and final [${\Psi }^{f}$] wave functions is given by $D({\boldsymbol{k}})=\langle {\Psi }^{f}({\boldsymbol{k}},{\boldsymbol{r}})|\nabla |{\Psi }^{i}({\boldsymbol{k}},{\boldsymbol{r}})\rangle$ and it is derived from the gradient of the Hamiltonian operator. Generally, the optical properties are evaluated with the frequency dependent of complex dielectric function as ε(ω) = ε_1_(ω) + iε_2_(ω). The imaginary part ε_2_(ω) of dielectric function could be obtained from the momentum matrix elements between the occupied and unoccupied wave functions, within selection rules as^34^:

$${\varepsilon }_{2}(\omega )=\frac{2{e}^{2}\pi }{\Omega {\varepsilon }_{0}}\,\sum _{{\boldsymbol{k}},c,v}\,{|\langle {\Psi }_{{\boldsymbol{k}}}^{{c}_{n}}|{{\boldsymbol{P}}}^{\alpha }|{\Psi }_{{\boldsymbol{k}}}^{{v}_{n}}\rangle |}^{2}\delta ({{\rm{E}}}_{{\boldsymbol{k}}}^{c}-{{\rm{E}}}_{{\boldsymbol{k}}}^{v}-E)$$

Ω is the volume of the supercell and ${\varepsilon }_{0}$ is the dielectric constant of free space. c and v represent the conduction band and the valence band, respectively. The imaginary part ε_2_(ω) is calculated by summing all transitions from occupied to unoccupied states over the Brillouin zone, weighted with the matrix element giving the probability of a transition. The real part ε_1_(ω) is then calculated by the Kramers-Kronig relation. The summations refer to the first Brillouin zone wave vectors.

### Thermal properties

The electrical and thermal conductivity can be understood in terms of transitions between energy bands. The thermal conductivity in terms of the spectral function of the Green’s function is given by the Kubo formula^33^:

$${\sigma }_{\mu \nu }({\rm{T}})\,=\frac{\hslash {{\rm{\sigma }}}_{0}}{{(2\pi )}^{2}}{\int }_{-\infty }^{\infty }\,[\frac{\partial f(\varepsilon ,T)}{\partial \varepsilon }]d\varepsilon \,{\int }_{FBZ}\,Tr[{\hat{v}}_{\mu }\hat{A}({\boldsymbol{k}},\varepsilon ){\hat{v}}_{\nu }\hat{A}({\boldsymbol{k}},\varepsilon )]\,d{\boldsymbol{k}}$$

${\hat{v}}_{\mu }$ is the velocity of electron along a ${x}_{\mu }$ direction, ${\rm{\omega }}$ is a frequency of incident photon and $f(\varepsilon ,T)={[1+exp(\varepsilon /{k}_{B}T)]}^{-1}$ denotes a Fermi-Dirac distribution function. The spectral function ${\boldsymbol{A}}({\boldsymbol{k}},\varepsilon )$ is related to the density of states^35^. The matrix components for ${\hat{v}}_{\mu }$ in the terms of **k** representation can be written as ${[{\hat{v}}_{\mu }]}^{ii}={\partial }_{{k}_{\mu }}{E}_{i}({\boldsymbol{k}})$. The paramagnetic susceptibility and electronic heat capacity is defined by the following expressions^36^:

$$\begin{array}{rcl}{\rm{C}}({\rm{T}}) & = & {\int }_{-\infty }^{\infty }\,\varepsilon [\frac{\partial f(\varepsilon ,T)}{\partial T}]D(\varepsilon )d\varepsilon \\ {\rm{\chi }}({\rm{T}}) & = & {\int }_{-\infty }^{\infty }\,\varepsilon [\frac{\partial f(\varepsilon ,T)}{\partial \varepsilon }]D(\varepsilon )d\varepsilon \end{array}$$

where the D(ε) is the DOS of the system. The electronic contribution of thermal conductivity can be measured from the following equation^37^

$${\kappa }_{\mu \nu }(T)=\frac{{{\rm{k}}}_{B}^{2}}{T}[{\Lambda }_{22}^{\mu \nu }(T)-\frac{{\Lambda }_{12}^{\mu \nu }(T){\Lambda }_{21}^{\mu \nu }(T)}{{\Lambda }_{11}^{\mu \nu }(T)}]$$

which ${\kappa }_{\mu \nu }$ is the electronic thermal conductivity tensor and the respective transport coefficient are:

$${\Lambda }_{ij}^{\mu \nu }(T)=\frac{{{\rm{\sigma }}}_{0}}{{{\rm{e}}}^{2}}\,{\int }_{-\infty }^{\infty }\,{\varepsilon }^{i+j-2}[\frac{\partial f(\varepsilon )}{\partial \varepsilon }]d\varepsilon \,{\int }_{FBZ}\,Tr[{\hat{v}}_{\mu }\hat{A}({\boldsymbol{k}},\varepsilon ){\hat{v}}_{\nu }\hat{A}({\boldsymbol{k}},\varepsilon )]\,d{\boldsymbol{k}}$$

with spectral function ${\boldsymbol{A}}({\boldsymbol{k}},\varepsilon )=-\,2Im\hat{G}(k,\varepsilon )$ and velocity of the electron ${v}_{\mu }$ in the $\mu$ direction^38^. The thermoelectric efficiency is optimized by lowering the thermal conductivity while keeping electrical conductivity high, therefore, the ratio of thermal conductivity to electrical conductivity known as the Lorenz number^37^ and by investigating the behavior of the Lorenz number $[L=\tfrac{{\kappa }_{\mu \nu }}{T\,{\sigma }_{\mu \nu }}]$, it is expected to improve the thermal properties of materials^39,40^.

### Computational details

In this study, we used DFT calculations to obtain the parameters required for the tight binding model. The DFT calculations are performed using the Spanish initiative for electronic simulations with thousands of atoms (SIESTA) package^41^. All the calculations were carried out with a the double-zeta plus polarization atomic orbital (DPZ) basis set and for the exchange and correlation terms, the Local Density Approximation (LDA) according to the Ceperly and Alder (CA) parameterization is used^42^. The cutoff energy for the plane waves is set to 450 Ry and all atoms were fully relaxed until the residual forces were smaller than 0.02 eV/Å. The atoms were allowed to relax until the residual forces were smaller than 0.01 eV/Å. A minimum vacuum distance of 20 Å between neighboring images is used to avoid the interaction between the adjacent layers. The Brillouin zone integration is performed by using the Gamma-centered Monkhorst-Pack scheme with 30 × 30 × 1 k-points the mesh of 90 × 90 × 1 k-points have been used for optical calculations.

## Results and Discussions

### Electronic structures and dipole matrix elements

**Figure 1:**
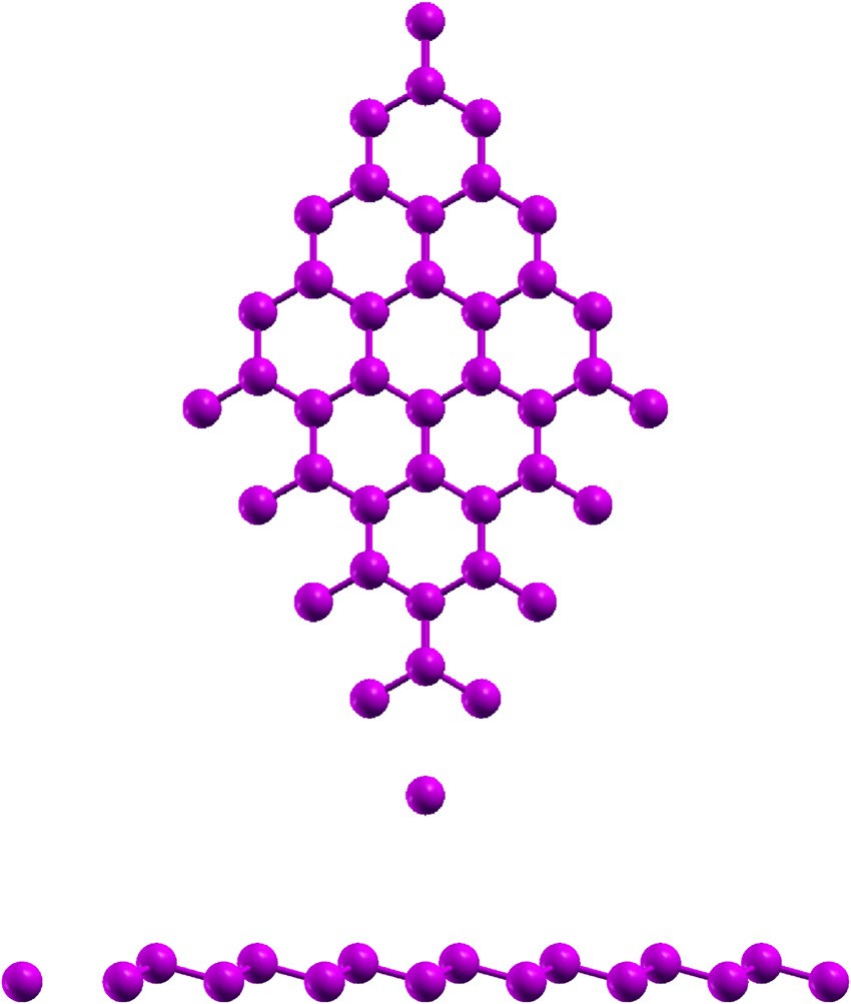
The geometrical structure of Germanene.

The optimized atomic structure of the Germanene with lattice constant of a = 3.97 Å and the Ge–Ge bond length 2.38 Å is shown in Fig. 1. The primitive unit cell include two Ge atoms.

**Figure 2:**
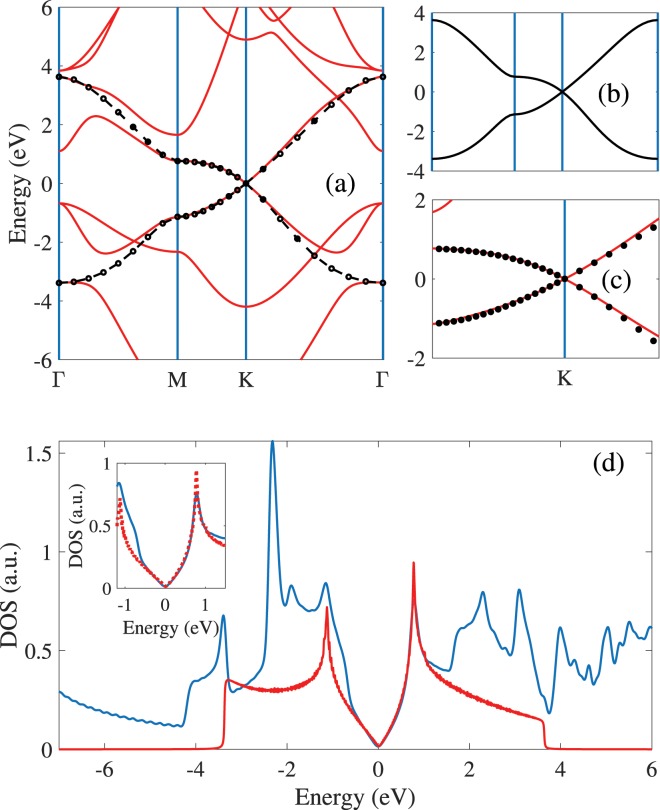
(**a**–**c**) Band structures of Germanene in the high Brillouin zone path ΓMKΓ. Red lines show DFT calculations and the black lines and dotted points depict the band structures from tight binding model. The Tight binding model shows that always two bands cross Fermi level at K-point. (**d**) DOS of Germanene structures with DFT and 3NN- tight binding calculations. The linear dispersion properties in band structure is found in the low energies of the DOS spectrum.

Figure 2(a–c) show the calculated band structures of monolayer Germanene along the high symmetric path in the Brillouin zone with the DFT [red lines] and tight binding [dotted lines] calculations. The Germanene has buckling due to the sp2/sp3 hybridazion which leads to out of plane height (Δz) for Ge atoms in their primitive unit cells. The calculated buckling height for Germanene is 0.65 Å which is in agreement with previous studies^14,30,43^. It can be observed that the highest unoccupied molecular orbital (LUMO) and lowest unoccupied molecular orbital (HOMO) have linear band dispersion in terms of momentum in vicinity of K-point and cross Fermi level at K point. Germanene has zero band gap and massless Dirac fermions. All these results for Germanene are in agreement with previous studies^18,44^. The dotted points in Fig. 2(a–c) show band structure of Germanene from tight binding model which determined by fitting with the DFT calculations. The required parameters are shown in Table 1. The Tight binding results are in good agreement with DFT and shows (i) linear properties of valence and conduction bands near the K-point and Fermi level and (ii) crossing valence and conduction bands and zero gap at K-point. Note that, the linear dispersion in K-point is due to the equal on site energies for both Ge atoms in the primitive unit cell. Finally, by comparing the ${{\boldsymbol{\nabla }}}_{{\boldsymbol{k}}}{\bf{E}}({\bf{k}})$ around Dirac point for graphene and Germanene structures, it can be see that the Fermi velocity of graphene is higher than that of Germanene. This can be explained by larger lattice constants and smaller hopping for Germanene in comparing with graphene.

For further study, we investigate the DOS of Germanene by tight binding model and DFT calculation. We used 3NN tight binding model to investigation the DOS of Germanene and comparison with DFT results, as shown in Fig. 2(d). DOS obtained from tight binding model has two Van Hove Singularities (VHSs) in the conduction and valence area on the both side of the Fermi level and in energies correspond to the lowest states in the band structure. The DOS is zero at Fermi level and increases linearly until reaches to peaks in both side of Fermi level. In this energy range, the linear increasing of DOS in terms of energy is due to the linear dispersion properties of band structure around the Fermi level and K- point. The distance between both VHSs is about 1.9 eV. The tight binding model shows that Germanene has the shoulder structures at higher energies which its origin could be linked to the band edge states at the Γ point. As shown in inset Fig. 2(c), both DFT and tight binding model show exactly the same results in the low energies of DOS spectrum of Germanene and this shows that our tight binding parameters are suitable for calculation the DOS of Germanene.

**Figure 3:**
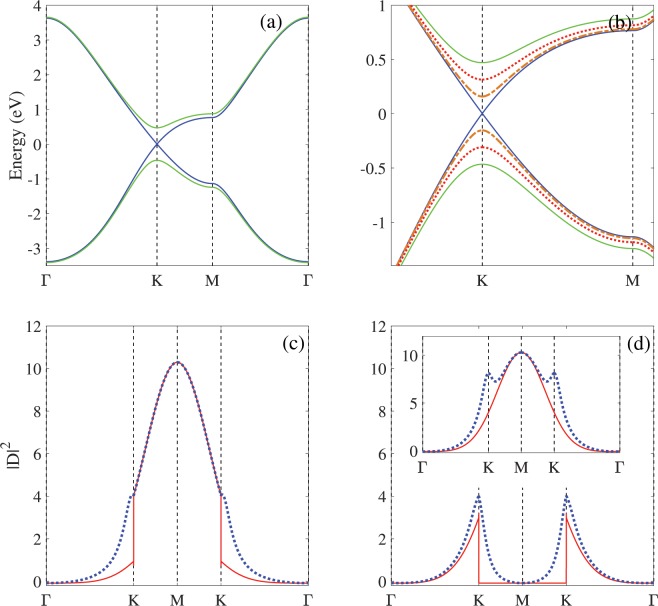
Band structures in the presence of bias voltage (**a**) in the high Brillouin zone path ΓMKΓ and (**b**) around the K-point. Near the K point and Fermi level, the linear bands in U = 0 change to the parabolic bands in U ≠ 0. The optical dipole of Germanene in (**c**) x- and (**d**) y- directions. Inset (**d**) show total dipole in same path.

One of the important aim in this work is investigation the optical properties of Germanene and for this purpose the dipole matrix elements should be calculated. Figure 3(a–d) show electronic properties and dipole matrix elements of Germanene with the tight binding model in the presence of bias voltages. Adding the potential parameters ±U/2 to the diagonal Hamiltonian matrix elements leads to appear the bias voltage to the Germanene system. By applying the bias voltage, because to electrical potential difference between two Ge atoms in the unit cell, the Ge atoms become inequivalent and their degeneracy at K point are removed, so the HOMO and LUMO bands move away from Fermi level. Also the VBM and CBM which are linear in the U = 0, change to parabolic bands in the presence of bias voltage (U ≠ 0) in the vicinity the K point [Fig. 3(a)]. In the presence of bias voltage, Germanene becomes semiconductor with direct band gap and the band gap shows linearly increases linearly with increasing the bias voltage. These results are in agreement with DFT results^23^. The focused band structure of Germanene in the KM path around the Fermi level and in the presence of different bias voltages is shown in the Fig. 3(b). The effects of bias voltage are significant around the K point than that others.

Figure 3(c,d) show the dipole matrix elements in terms of wave vector k in the ΓKMKΓ path for x and y directions (|D_x_|^2^ and |D_y_|^2^), in the different bias voltages. The 3NN- interactions are included to calculate the momentum dependence of dipole matrix elements. The x- direction shows stronger dependence to the wave vector k than that y- direction. Our results show quadratically increasing for |D|^2^ in ΓK path for both x- and –y directions. In the KM path the |D|^2^ shows different behaviors for x- and y- directions. In this path the |D_x_|^2^ is increased to its maximum value but |D_y_|^2^ is decreased to zero. The |D_x_| has maximum value at M point and the |D_y_| has maximum value at K point. Inset Fig. 3(d) shows total dipole |D|^2^ in the same path. In the presence of bias, the |D_x_|^2^ obviously increases at ΓK path with same value in the K point and also it remains unchanged in KM path. The applied bias voltage changes the behavior of |D_y_| in the KM path and it becomes non zero in this path. Also the |D_y_| value increases with bias in the K- point.

### Optical properties

**Figure 4:**
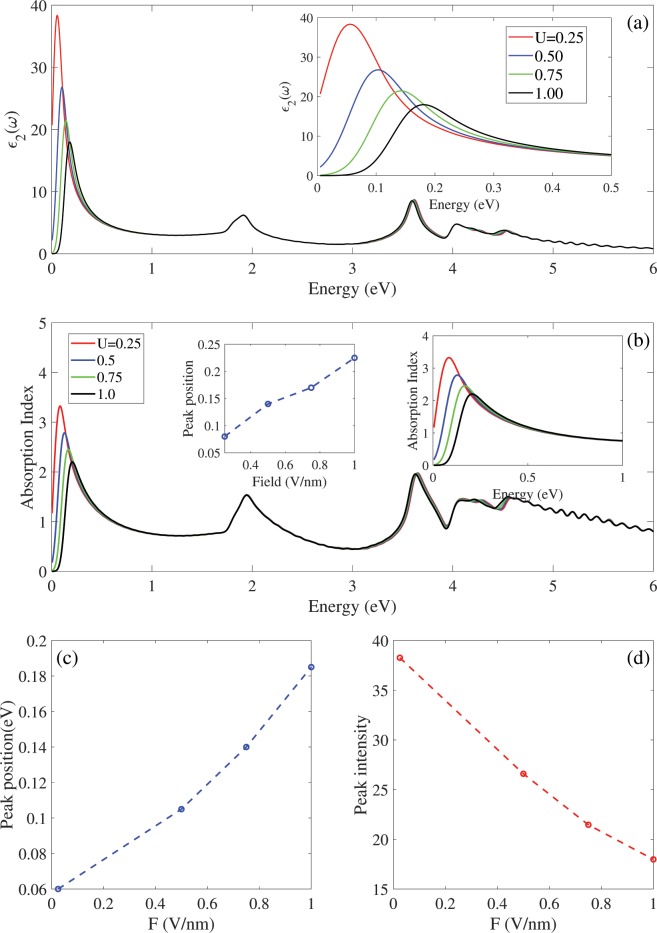
The DFT result for (**a**) imaginary parts of dielectric function and (**b**) absorption spectrum in the presence of electric field. Insets show spectra in in low energies. Left inset (**b**) show absorption peak position in terms of electric field. (**c**,**d**) Show positions and intensities of imaginary parts of dielectric function in terms of electric field.

Figure 4 shows the imaginary part of dielectric function and absorption spectrum of Germanene in the presence of vertical electric fields with DFT calculation. Both spectra have three distinct peaks in E < 1 eV, E ≈ 1.9 eV and 3 < E < 5 eV, region independent to the electric field. Both spectra show same peak position and peak displacement in the presence of electric field. The electric field significantly affected the first peak in the E < 1 eV due to the effects of electric field on the band structure near the Fermi level around the K point. As shown in Fig. 4(a,b), the electric field shifts the first peak to higher energy and reduces its intensity due to the increasing the band gap in presence of electric field. The electric field has not affected the positions and intensities of other peaks in E > 1 eV region. As shown in Fig. 4(c,d), the regular pattern is found for position and intensity of the first peak in terms of the electric field. When vertical electric field is applied to the Germanene, the first peak has linear displacement with decreasing the intensity.

**Figure 5:**
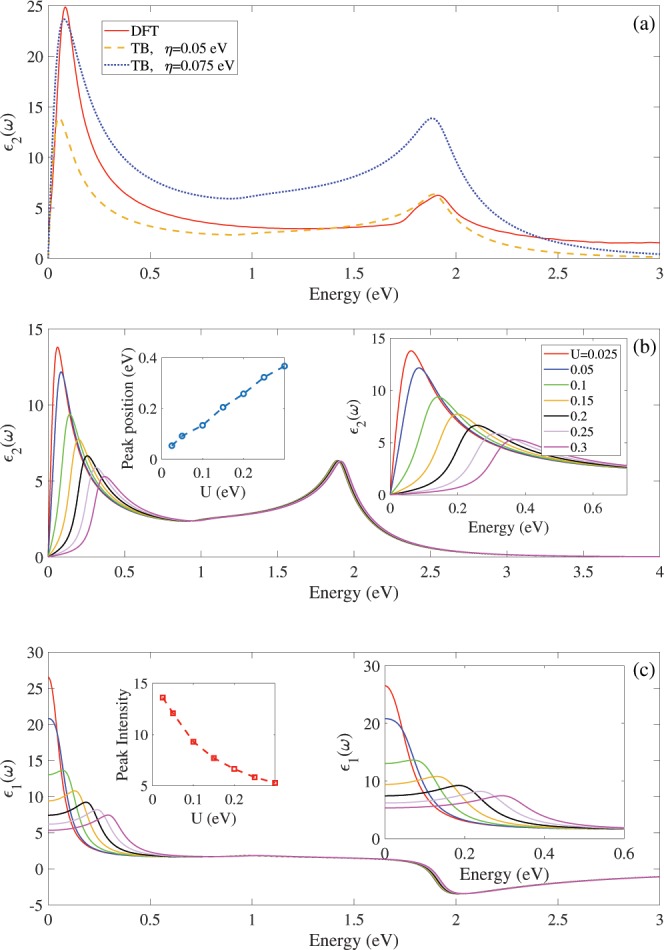
(**a**) The tight binding based DFT calculation of dielectric function of Germanene up to visible frequency ћω < 3 eV, with two coefficients η = 0.05 and 0.075 eV for comparison the Tight binding and DFT results. (**b**) Imaginary and (**c**) real parts of dielectric function in the presence of bias voltages. The blue shift increases by the increasing the bias strength due to the increasing of the band gap of Germanene. Left insets (**b**,**c**) show positions and intensities of dielectric function with tight binding model. Right insets (**b**,**c**) show spectra in in low energies.

Now the influence of electric field on the real and imaginary parts of dielectric function is investigated via the tight binding based on our DFT calculation in the visible energies ћω < 3 eV, as shown in the Fig. 5. To compare the tight binding results with the DFT, we investigate the tight binding imaginary parts of dielectric function with two broadening coefficients ${\boldsymbol{\hslash }}{\rm{\eta }}{\rm{=}}{\rm{0}}.{\rm{05}}$ and 0.075 eV [Fig. 5(a)]. The optical spectrum with both coefficients have similar optical peak positions with different intensities and these results are strong enough to ensure the validity of our tight binding parameters. For the ${\boldsymbol{\hslash }}{\rm{\eta }}{\rm{=}}{\rm{0}}.{\rm{05}}$ (0.075) coefficients, the intensity of the second (first) peak is identical with the DFT results.

Figure 5(b,c) show effects of the bias voltage on the imaginary and real parts of dielectric function, using the tight binding model. The results show that by applying and increasing the bias voltage, the first peak position in the infrared region, shifts to higher energies with the peak intensity reduction. Note that, the blue shift increases by the increasing the bias voltage due to the increasing of the band gap. The position of first peak in 0.065 eV at U = 0.025 eV moves to 0.367 eV at U = 0.1 eV [right inset Fig. 5(b)]. The displacement of the first peak position has a linear relationship with the bias strength [left inset Fig. 5(b)]. The intensity of the first peak decreases linearly with bias strength [left inset Fig. 5(c)]. The position and intensity of the second peak in the visible region does not show obviously change with applying and increasing the bias voltage. This peak is associated with van Hove singularities in the density of states. Finally, as shown in Fig. 5(c) the values of static dielectric constant decreases with increasing the bias voltage.

### Thermal properties

**Figure 6:**
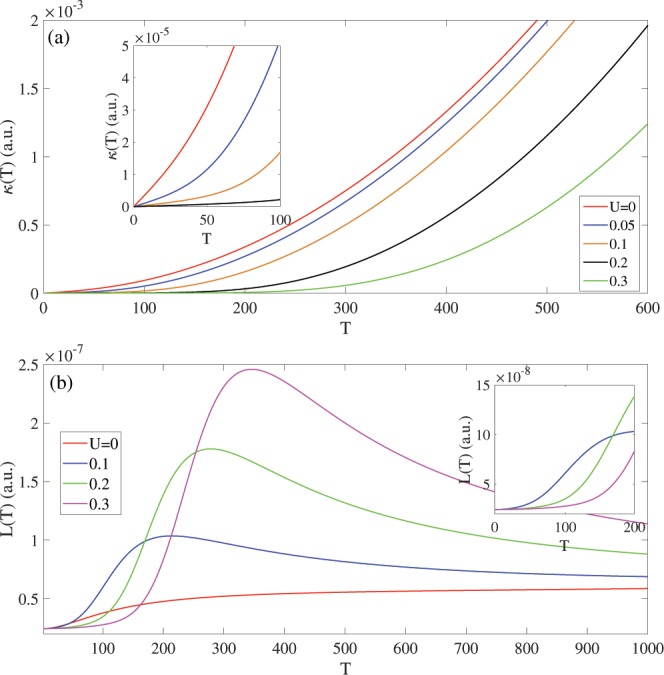
The thermal conductivity of Germanene in the presence of bias voltage in (**a**) T < 600 K. The k(T, U) increases with temperature for any bias strength. (**b**) The Lorenz number in the presence of bias voltage and T < 1000 K. Larger bias voltage leads to higher Lorenz number.

**Figure 7:**
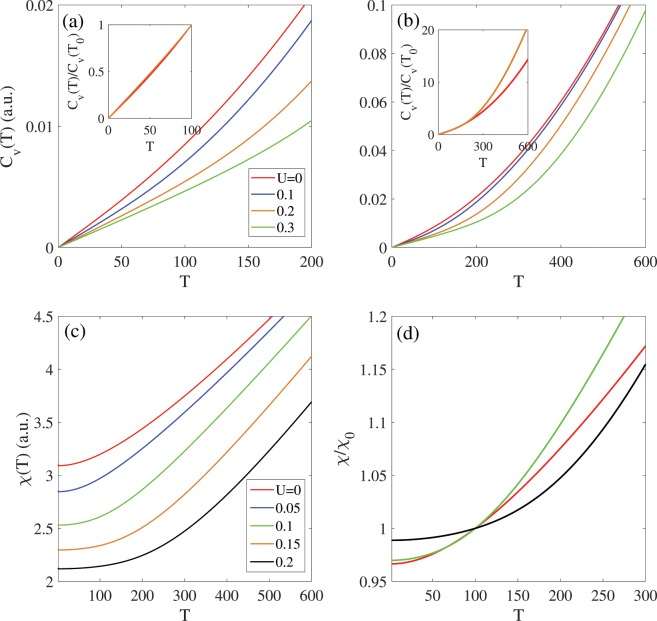
The heat capacity in (**a**) T < 200 K and (**b**) T < 600 K, in the different bias voltages. Heat capacity increases with temperature independent to the bias strength. Inset shows the heat capacity per unit C0(T0 = 100 K). The C(T, U)/C0 for all biases are equal in T < 100 K. (**c**) The temperature dependence of paramagnetic susceptibility of Germanene in different bias strength. The paramagnetic susceptibility in U ≠ 0 is smaller than that U = 0 T < 600, due to the band gap behavior. (**d**) The χ(T, U) per χ_0_(T0 = 100, U) for U = 0, 0.1 and 0.2 eV.

In this section, the temperature dependence of various thermoelectric functions are investigated in the presence of bias voltages. Figures 6 and 7 show the electronic thermal conductivity [κ(T)], the Lorenz number [L(T)], the heat capacity [C_v_(T)] and paramagnetic susceptibility [χ(T)] in terms of the temperature [in range T < 600 K].

We begin our investigation of Germanene with a discussion of the thermal conductivity k(T) in temperature range T < 600 K. The similarities and differences of biased and un-biased Germanene is shown in Fig. 6(a). The k(T) in terms of temperature increases without dependence on the presence or absence of bias voltage. The reason for this behavior is increasing the thermal energy kbT of the charge carriers with temperature increasing. The k(T, U = 0) is larger than that k(T, U ≠ 0) in temperature range T < 600 K and the decreasing κ with bias voltage arises from increased band gap in presence of bias voltage. Also the k(T, U) decreases by further bias increasing because the bias voltage behaves as a potential barrier to excitation the charge carriers and they have not enough energy for transition between level states. In the low temperature range, the k(T, U) can be described with k ≡ νΔT, where ν is increasing rate and it decreases with increasing the bias strength.

Figure 6(b) shows behavior of Lorenz number L(T) in terms of temperature and different biases. In low temperature L(T) is close to zero, independent to the bias strength. By further temperature increasing, the L(T) increases to its maximum value then decreases. To enhanced further the L(T) strength, its necessary to applying and increasing the bias voltage. By applying the bias voltage, the intensity of L(T) increases in all temperature range. The stronger bias voltage leads to larger L(T) and for all bias voltage below U < 0.3 eV, L(T) has a peak in the T = Tc in T < 600 K. The position of peak shifts to higher temperature with increasing the bias voltage.

Figure 7(a,b) shows heat capacity Cv(T) of Germanene in the low and high temperature region and different bias voltage. The heat capacity is zero in the T = 0 K and it increases with temperature increasing independent to the bias voltage. In the low-temperature range T < 200 K, this increasing is linearly proportional to the temperature as Cv(T)∝T and in higher temperature range Cv(T) increases parabolically with temperature. The Cv(T ≠ 0, U) is smaller than that Cv(T = 0, U) due to the band gap opening in the presence of bias voltage. The increasing rate of Cv(T) in terms of T decreases with increasing the bias voltage. For more study to obtain results independent of the bias strength, we investigate the heat capacity per unit C_0_(T = 100 K). In the lower temperature range T < 100 K, the C(T)/C_0_ are equal for applied biases in U < 0.3 eV range, and by further increasing the temperature [T > 100 K] their differences increased as shown in the inset Fig. 7(b). In the higher temperature range, the stronger bias has larger C(T)/C_0_ than that smaller U.

Figure 7(c) shows the temperature dependence of paramagnetic susceptibility [χ(T, U)] in different bias voltage. In the T < 600 K, the χ(T, U) increases by increasing the temperature and this behavior remains unchanged in presence of bias voltage. In U ≠ 0, the χ(T, U) under smaller bias strength is larger than that stronger bias. As shown in Fig. 7(d), by investigation the χ(T, U) per χ_0_(T0 = 100, U), it is found that the in the T < 100 K, increasing rate for U = 0.2 eV is larger than that U = 0 and 0.1 eV. By increasing the temperature in the 100 < T < 300 K range, the χ/χ_0_(U = 0.1) > χ/χ_0_(U = 0) > χ/χ_0_(U = 0.2).

## Outlook and Conclusion

In summary, using DFT calculation and the tight binding model, we calculate the electronic properties, dipole matrix elements and optical spectrum of monolayer Germanene. Also, using the Green’s function method, the thermal conductivity, heat capacity and paramagnetic susceptibility of Germanene has been calculated in terms of temperature and in the presence of applied bias voltage. The required tight binding parameters are obtained based on DFT calculations. The band structure of Germanene shows linear behavior in terms of wave vector in vicinity of K point at Fermi level and it is semiconductor with zero band gap. Germanene shows band gap opening in presence of bias at K point and its band gap increases linearly with increasing the bias strength. For investigation the optical properties of Germanene, the dipole matrix elements |D|^2^ investigated. It is found that in the KM path, the|Dx|^2^ is increased to its maximum value but |Dy|^2^ is decreased to zero. By applying the bias voltage, the |Dx|^2^ increases in the ΓK path and remains unchanged in the KM path. Also the strength of dipole in the y-direction is smaller than that x-direction. The tight binding model and DFT calculations show similar behavior for optical spectra including the same peak positions. Our results show that in the presence of bias voltage, the first optical peak in the infrared region moved to higher energies with intensity reduction due to band gap opening for Germanene in U ≠ 0. The thermal properties of Germanene in terms of temperature increases without dependence on the presence or absence of bias due to an increased thermal energy kbT of the charge carriers with temperature. The k(T) and C_v_(T) in U = 0 are larger than that U ≠ 0 in temperature range T < 600 K due to increasing the band gap in presence of bias voltage. In the low temperature range, the κ(T) and C_v_(T) can be described linearly with temperature [κ ≡ ν_1_ΔT and C_v_ ≡ ν_2_ΔT] where ν_1_ and ν_1_ are the increasing rate and they decrease by bias strength. Although the required tight binding parameters for Germanene have been obtained, in agreement with the DFT results, but there are still many aspects that need further study. This model can be used to investigation the electronic/thermal/optical properties of Ge-nanoribbons and Ge-nanotubes. Also, using non-equilibrium Green’s function technique based on tight binding approach, this model can be used for investigation effects of atom dopant and molecules adsorption on various 1 and 2 dimensional Germanene systems such as mono/bilayer, nanotubes and nanoribbons.
